# Substance P as a potential salivary biomarker to differentiate Sjögren’s disease from non-Sjögren sicca syndrome: a pilot study

**DOI:** 10.3389/fimmu.2026.1851367

**Published:** 2026-06-09

**Authors:** Pamela Rosso, Elena Fico, Annamaria Di Criscio, Roberta Priori, Emanuele Grilli, Filippo Valentini, Sarah Amdu Retta, Paola Tirassa, Marco Fiore, Massimo Fusconi, Cinzia Severini

**Affiliations:** 1 Institute of Biochemistry and Cell Biology, National Research Council (CNR), Rome, Italy; 2 Ma.CRO Lifescience Srl, Roma, Italy; 3 Department of Sense Organs, Sapienza University of Rome, Rome, Italy; 4 Unità Operativa Complessa Rheumatology, Department of Internal Medicine and Medical Specialties, Azienda Ospedaliero-Universitaria (AOU) Policlinico Umberto I, Rome, Italy

**Keywords:** biomarker, saliva, sicca, Sjögren’s disease, substance P

## Abstract

**Objective:**

The diagnosis of Sjögren’s Disease (SjD) remains challenging due to the non-specific nature of sicca symptoms and the need for invasive biopsies. This pilot study aimed to investigate the potential of salivary Substance P (SP), a neuropeptide involved in glandular regulation and neurogenic inflammation, as a novel, non-invasive biomarker to differentiate patients with SjD from those with non-autoimmune Non-Sjögren sicca syndrome (NSS).

**Methods:**

Unstimulated whole saliva samples were collected from three groups of female participants: 13 patients classified as SjD according to ACR/EULAR criteria, 13 patients with idiopathic NSS symptoms who did not meet the criteria, and 13 healthy controls (CTRL). Salivary SP concentrations were measured using a specific enzyme-linked immunosorbent assay (ELISA). Clinical, serological, and histopathological data were recorded for correlation analyses. Receiver operating characteristic (ROC) curve analysis was used to evaluate the diagnostic performance of salivary SP.

**Results:**

Salivary SP levels were significantly higher in SjD patients (mean 92.2 ± 15.503 pg/ml) compared to both NSS patients (30.39 ± 4.08 pg/ml, p < 0.005) and healthy controls (39.93 ± 5.97 pg/ml, p < 0.005). No significant difference was found between the NSS and CTRL groups. ROC analysis demonstrated that salivary SP could distinguish SjD from NSS, with an area under the curve (AUC) of 0.8225 (p = 0.005). At a cut-off value of 47.63 pg/ml, sensitivity was 77% and specificity was 85%. A positive association was observed between salivary SP levels and the presence of ANA autoantibodies in the SjD cohort.

**Conclusion:**

Salivary SP appears to be elevated in patients with SjD compared to those with NSS in this preliminary study, suggesting a potential diagnostic signal that warrants further investigation. Although the observed differences hint at possible utility as a non-invasive biomarker related to neuroimmune dysregulation, the diagnostic accuracy observed should be interpreted with caution due to the limited sample size and the exploratory nature of the findings. Larger, prospective, and well-powered studies are needed to confirm these preliminary observations, to assess the robustness of salivary SP as a diagnostic tool, and to determine its potential clinical applicability.

## 1 Introduction

Sjögren’s Disease (SjD) is a chronic, systemic autoimmune disorder characterized by progressive dysfunction and inflammation of the exocrine glands, most notably the lacrimal and salivary glands, leading to impaired secretory function (1). Beyond these cardinal symptoms, SjD can also present with a wide range of systemic manifestations, including fatigue, arthralgia, and involvement of various organ systems such as the pulmonary, renal, and neurological systems (2). Women are mostly affected (female to male ratio = 9:1), with the majority of cases being diagnosed in the 5th or 6th decade of life (1). According to the widely accepted American-European Consensus Group (AECG) criteria and the subsequent American College of Rheumatology/European League Against Rheumatism (ACR/EULAR) classification criteria, a definitive diagnosis typically requires the presence of focal lymphocytic sialadenitis in a minor salivary gland biopsy, and/or the presence of specific autoantibodies such as anti-Ro/SSA and/or anti-La/SSB, and at least objective evidence of reduced salivary and lacrimal gland function (3–5). Despite these established criteria, the diagnostic process for many patients is challenging and often delayed (6). SjD shares symptoms of glandular dysfunction with non-Sjögren sicca syndrome (NSS), which is frequently reported in the general population and is often associated with ageing, medication use, or other medical and autoimmune conditions (7, 8). Given the complexity of diagnosis (9), there is an urgent and unmet need to identify novel, non-invasive, disease-specific biomarkers.

The analysis of biological fluids, particularly saliva, offers a promising and attractive approach (8, 10, 11). As the direct product of the target organ in SjD, saliva reflects the local inflammatory and physiological milieu of the salivary glands, providing a unique window into disease processes without the need for invasive procedures (12, 13). The pathogenesis of salivary gland dysfunction in SjD is multifactorial, involving not only lymphocytic infiltration and destruction of glandular tissue but also significant neurogenic dysregulation. It has been demonstrated that patients with SjD exhibit impaired innervation of the salivary glands, along with alterations in circulating levels of neuropeptides, which are thought to contribute directly to the observed hyposalivation (13–15). It is postulated that autoimmune and inflammatory processes may lead to vaso-neural dysregulation and peripheral nerve injury, ultimately resulting in decreased fluid secretion and duct cell atrophy (16). Neuropeptides, including Neuropeptide Y (NPY), Vasoactive Intestinal Peptide (VIP), Calcitonin Gene-Related Peptide (CGRP), and Substance P (SP), are co-released with classical neurotransmitters and play a crucial role in modulating salivary gland function, significantly stimulating salivary flow (17–19). Among these neuropeptides, SP, a member of the tachykinin family, in addition to its neurotrophic and neuroprotective activity in the central (CNS) and peripheral nervous systems (PNS) (20–23), is involved in glandular regulation (24).

Furthermore, SP is a component of saliva, and its function may be to promote the repair of ongoing lesions in the oral mucosa, as its role in accelerating wound healing in various tissues is well established (25–27). In salivary glands, SP-immunoreactive nerve fibers are mainly localized around blood vessels and in direct contact with acinar cells, enabling them to influence both blood flow and secretory activity (28). Notably, the number of these nerve fibers is significantly reduced in the salivary glands of SjD patients (14). However, no data were available on the concurrent expression and distribution of its high-affinity receptor, Neurokinin-1 Receptor (NK1R). This gap was addressed by recent research from our group, which revealed significant dysregulation of the SP/NK1R system in the salivary glands of SjD patients. This study demonstrated a marked reduction in SP expression alongside compensatory upregulation of NK1R in glandular tissue compared to control subjects with non-autoimmune NSS symptoms (29). To date, no clinically validated, disease-specific biomarkers have been successfully incorporated into the classification criteria for SjD. To address this gap and translate our histological findings into a potential clinical tool, this study was designed to investigate the involvement of this neuropeptide in a more accessible biological fluid. The primary aim was to analyze the concentration of SP in the saliva of patients with SjD, comparing these levels to those in individuals with non-autoimmune NSS symptoms and healthy control subjects. We aimed to determine whether salivary SP could serve as a potential, non-invasive, disease-specific biomarker for SjD.

## 2 Materials and methods

### 2.1 Patients’ selection and enrollment

Saliva samples were collected from patients with suspected SjD who were followed up in our dedicated Sjögren’s Clinic at Sapienza University (Department of Clinical Internal, Anesthesiological and Cardiovascular Sciences – Division of Rheumatology). Recruitment was prospective, and patients were enrolled consecutively. Only female participants were enrolled because SjD has a strong female predominance (female:male ratio ≈ 9:1), and sex-related differences in neuropeptide levels are well documented (30). The mean age of the suspected SjD (NSS + SjD) patients was 53.31 ± 2.49 years (n=26). The mean age of the age-matched healthy controls (CTRL) was 56.62 ± 3.46 years (n=13). After reviewing the diagnostic criteria, patients were classified as having SjD if they fulfilled the ACR/EULAR criteria (total score ≥4); those who did not meet the classification criteria were identified as having NSS (3). According to these criteria, out of the 26 suspected SjD patients, 13 were diagnosed with SjD and 13 were classified as NSS. These 13 SjD patients, 13 NSS patients, and the 13 CTRL were subsequently analyzed. Exclusion criteria were a) ongoing treatment with corticosteroids, hydroxychloroquine, immunosuppressants, or pilocarpine within the previous 3 months, b) periodontal disease or oral inflammation (patients with active gingivitis or periodontitis were excluded by clinical oral examination at the time of saliva collection), c) use of drugs that could influence the serum levels of SP, such as beta-blockers and d) as neuropeptide levels are affected by stress, pain, or neurological conditions, patients with fibromyalgia, neuropathy or chronic pain syndromes or those treated with antidepressants, antipsychotics and opioids were excluded.

Patient characteristics are described in detail in **Table 1**.

**Table 1:** Demographics and clinical characteristics of the cohort.

Variable	SjD (n = 13)	NSS (n = 13)	CTRL (n = 13)	P
Median age (yr)	60 ± 2.83	46.6 ± 3.2	56.62 ± 3.46	
ANA positive	9/13	2/13		
ENA positive	2/13	1/13		
SCHIRMER positive	10/13	7/13		
Median biopsy focus score (FS)	2.57 ± 0.4			
ESSPRI	4.73 ± 0.6			
ESSDAI	0.83 ± 0.4			
SP (pg/ml)	92.2 ± 15.503	30.39 ± 4.08	39.93 ± 5.966	<0.001

### 2.2 Ethical statement

The study was conducted in accordance with the Declaration of Helsinki. Authorization for the use of biological samples for research purposes was obtained from the Institutional Review Board or Ethics Committee at Sapienza University of Rome under protocol number 4688. Written informed consent was obtained from all patients participating in the study. All personal information was anonymized for processing and storage in accordance with ethical requirements.

### 2.3 Sample collection

Saliva collection took place in the morning, generally between 8:00 and 9:00 AM (to control circadian variation), following a minimum two−hour fast. Additionally, tooth brushing and smoking were prohibited for two hours before collection. The procedure involved unstimulated saliva collection over 15 minutes. Patients were asked to lean their heads forward and let saliva flow into a sterile container without active mouth movements. Whole saliva specimens were immediately placed on ice and then kept at −80°C until they were analyzed.

### 2.4 Analysis of samples

The collected saliva samples were stored at –80 °C until used for ELISA analysis. To quantify the expression level of SP, an ELISA assay was performed using a commercial kit for human SP according to the manufacturer’s instructions (Human Substance P ELISA Kit, A80226, Abcam, Cambridge, UK). The test is based on the colorimetric quantification of the peptide present in saliva after an antigen-antibody reaction specific for SP, comparing the values obtained with those of a standard curve. All samples were analyzed in duplicate and performed blindly.

### 2.5 Statistical analysis

We performed statistical analyses using GraphPad Prism (version 7, GraphPad Software). All data are expressed as the mean ± standard error (SE). Normal distribution was analyzed with the Levene’s test and further revised by the Welch’s correction method. Power analysis according to SP was carried out by using the Sample Size Calculator “ClinCalc.com”. Statistical analysis was conducted using one-way ANOVA, corrected if necessary, by Welch’s ANOVA. Statistical significance was set at p < 0.05. Categorical variables were compared using the chi-square test. Correlations were evaluated with Pearson’s correlation test. Receiver operating characteristic (ROC) analyses were used to evaluate the SP test’s ability to discriminate between groups across all possible cut-off values.

## 3 Results

### 3.1 SP in saliva of CTRL, NSS and SjD patients

**Figure 1:**
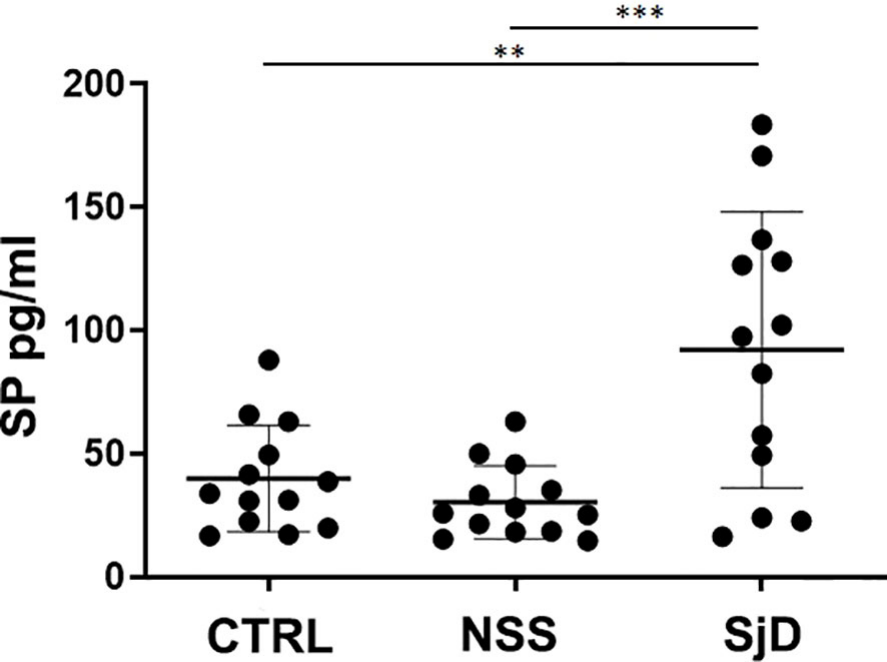
Saliva SP graph from SP ELISA kit. CTRL, Control Patients; NSS, Non-Sjögren sicca syndrome; SjD, Sjögren’s Disease Patients. **p<0.01; ***p<0.001. Statistical analysis was assessed by one-way ANOVA followed by Tukey’s post‐hoc test.

Salivary SP output (pg/ml) was significantly increased in SjD patients (92.2 ± 15.503) compared to NSS (30.39 ± 4.08) and CTRL patients (39.93 ± 5.966), p < 0.005 (**Figure 1**). In **Figure 1**, the graph shows the different distribution of saliva SP of CTRL, NSS and SjD patients, analyzed by the SP specific ELISA Kit. A statistical difference can be found when comparing the CTRL group and the SjD one (**p<0.01) or the NSS group with SjD (***p<0.001).

On the contrary, no significant difference in SP levels between NSS patients and CTRL has been found. SP findings adjusted by the age factor ANCOVA did not disclose a role of the woman age in these differences (p=0.308).

### 3.2 Power analysis

The Sample Size Calculator was carried out by “ClinCalc.com”, according to SP values. The Study Parameters were: Mean SP, population 40; Mean SP, study group 90; Alpha 0.05; Beta 0.2; Power 0.8.

### 3.3 ROC curve analysis of salivary SP in SjD, NSS, and CTRL patients

**Figure 2:**
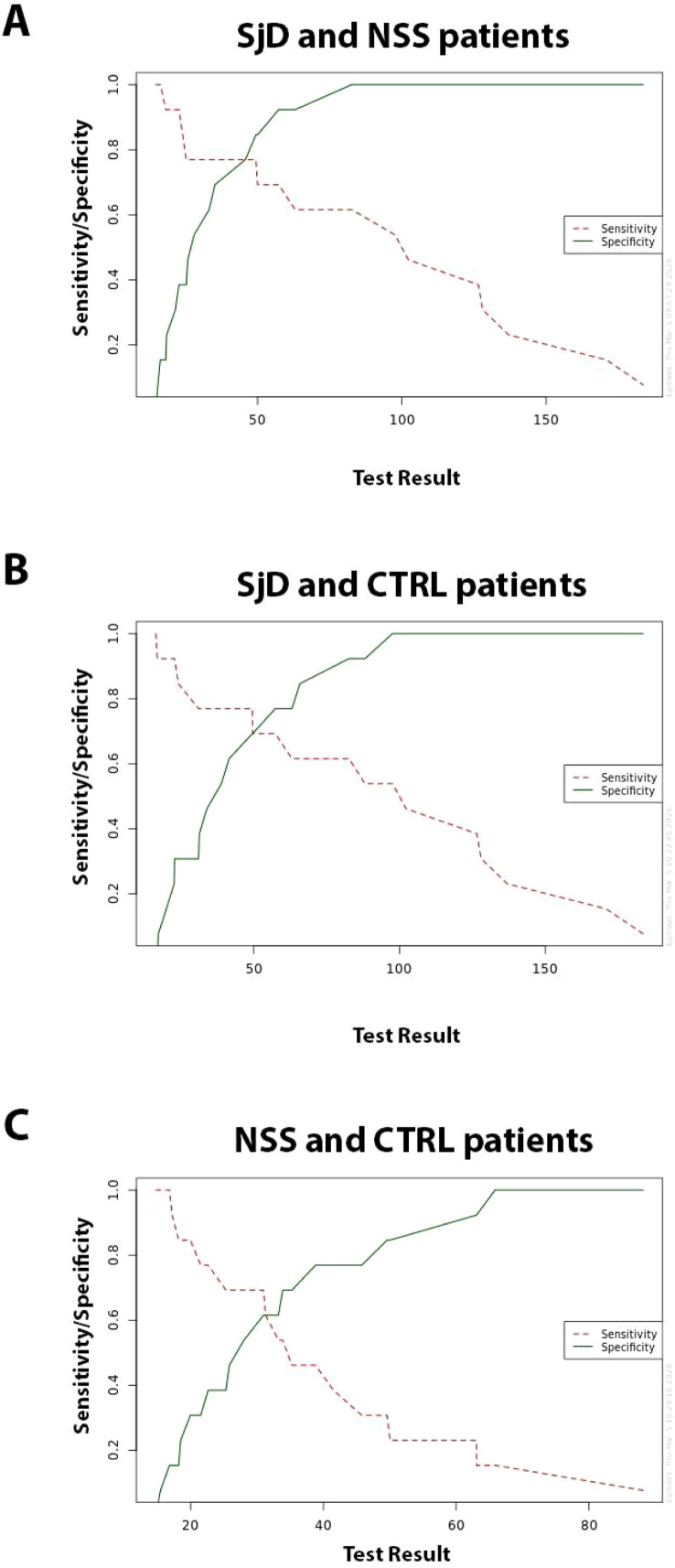
ROC curve between **(A)** SjD and NSS patients; **(B)** SjD and CTRL patients; **(C)** NSS and CTRL patients.

To assess the diagnostic utility of salivary SP, ROC curve analyses were carried out. For distinguishing SjD from NSS patients, the area under the curve (AUC) was 0.8225 (95% CI: 0.6497 to 0.9952, p = 0.005). A cut-off value of 47.63 pg/ml yielded a sensitivity of 77% and a specificity of 85% for this comparison. When comparing SjD patients with healthy controls, the AUC was 0.7692 (95% CI: 0.5741 to 0.9644, p = 0.02), with a cut-off of 45.49 pg/ml providing a sensitivity of 69.2% and a specificity of 76.9%. However, salivary SP could not discriminate between NSS patients and CTRLs, as indicated by a non-significant AUC of 0.633 (95% CI: 0.4113 to 0.8550, p = 0.25) (**Figure 2**).

Pearson’s correlation analysis revealed no significant correlations between salivary SP and clinical parameters in SjD patients, such as the EULAR SjD activity index (ESSDAI), EULAR SjD Patient Reported Index (ESSPRI), the SCHIRMER test, able to measure tear production, age and Focus Score index (FS).

## 4 Discussion

The diagnosis of SjD, particularly in patients who do not meet the ACR/EULAR criteria or who initially present with isolated visceral involvement, remains a clinical challenge (31). This diagnostic uncertainty often results in a significant delay, with an estimated interval of 6 to 10 years between the onset of symptoms and final diagnosis (30, 32). Although minor salivary gland biopsy is a keystone of the classification criteria, its invasive nature or inconclusive results highlight the urgent need for reliable, non-invasive biomarkers that can accurately distinguish SjD from NSS.

Our primary finding is that salivary SP concentrations are significantly higher in patients with SjD than in those with NSS. This elevation suggests a potential tool for distinguishing SjD from NSS patients, as demonstrated by ROC analysis. At a cut-off value of 47.63 pg/ml, salivary SP showed a sensitivity of 77% and a specificity of 85% suggesting it may represent a practical support for this challenging differential diagnosis. Despite the limited sample size, we also explored the potential predictive value of SP. Among the 13 patients with SjD, only two had salivary SP levels below the 47.63 pg/ml cut-off.

This interpretation is further supported by a closer examination of the outlier values within our NSS cohort. Although the mean salivary SP concentration did not differ significantly between the NSS and CTRL groups, we found that the SP values exceeding the cut-off in the NSS group were attributable to two specific patients, who were the only ones in the group positive for autoantibodies ANA or ENA; one of them was later diagnosed with Lupus. Therefore, it is reasonable to hypothesize that in a larger, more rigorously selected cohort, where all NSS patients with autoantibodies are excluded, the discriminatory power of salivary SP would be even greater.

The elevated levels of SP in saliva reported here might therefore appear paradoxical given the reduced glandular innervation. However, this could be interpreted in the context of neurogenic inflammation (33).

Indeed, two key alterations are observed in SjD. First, histological studies show a significant decrease in the density of SP-immunoreactive nerve endings in SjD glands, suggesting local denervation that could contribute to acinar atrophy (16). Second, and most relevant for neurogenic inflammation, the immune cells infiltrating the glands, particularly mast cells, plasma cells, and lymphocytes, begin to produce SP themselves. Approximately 46% of these immune cells in the glands of SjD patients are immunoreactive for SP, while such immunoreactivity is absent in CTRLs (14).

This ectopic SP production by immune cells creates a persistent neurogenic microenvironment: locally released SP can bind to NK1 receptors on the same immune cells or on nerve endings, amplifying inflammation, activating nociceptive pathways (contributing to the sensory neuropathy often described in SjD), and promoting atrophy, apoptosis, and necrosis (14). Moreover, the upregulation of NK1 receptor levels in the salivary glands of SjD patients can make the remaining tissue and cells hyper-responsive to the SP that is present, potentially amplifying its effects even at lower amounts (29).

Overall, the ectopic source of SP could lead to increased SP release in saliva, although direct evidence is lacking and should be addressed in future studies.

Thus, salivary SP may not simply reflect the density of innervation, but rather the functional state and pathological activation of the neurogenic pathway. This hypothesis is further supported by the absence of a significant difference in SP levels between NSS patients and CTRLs, which is an important negative finding, suggesting that the neuropeptide alteration is specific to the autoimmune inflammatory process of SjD (34). It supports the argument that elevated SP in SjD is not a nonspecific consequence of hyposalivation, which occurs in both SjD and NSS patients. Instead, it reinforces the concept that the dysregulation is intrinsically linked to the autoimmune pathogenesis characteristic of SjD (35).

This study successfully translates our previous histological observations of a dysregulated SP/NK1R system in SjD salivary glands into a clinically accessible, fluid-based biomarker (29).

The main strength is that dividing the groups (SjD vs NSS vs CTRL) allows the correct hypothesis to be tested in the appropriate clinical context, aiding in making a difficult diagnosis.

Salivary biomarkers are increasingly recognized as promising non-invasive tools for diagnosing of SjD (2). Although several classes of biomarkers have shown potential, their true discriminative ability remains uncertain when not tested against patients with NSS, which is essential for clinical applicability. For example, soluble siglec-5 (36) vasoactive intestinal peptide (VIP), and neuropeptide Y (NPY) (4), have only been compared with CTRLs, providing no evidence of their ability to distinguish SjD from NNS. In contrast, some individual biomarkers have demonstrated this capability. Salivary β2-microglobulin, for instance, showed an optimal cut-off of 0.472 ng/mL and was significantly elevated in SjD patients compared to NSS (5). Among cytokines, Th17-associated ones (TNF-α, IFN-γ, IL-22, and IL-17) were also significantly higher in SjD than in NNS (7). These results highlight that relying solely on CTRLs comparisons is inadequate for assessing a biomarker’s ability to differentiate SjD from NSS, a distinction crucial in clinical practice. Notably, our pilot study on SP yielded a cut-off value similar to this validated for β2-microglobulin in a large cohort, further supporting the role of salivary SP as a potential discriminative biomarker for SjD.

However, this study has several limitations that must be acknowledged. The most significant is the small sample size, which, although sufficient for a pilot investigation, limits the generalizability of the findings and the statistical power for subgroup analyses. The inclusion of only female participants, while justified by the strong female predominance of SjD and known sex-related differences in neuropeptide levels, represents a limitation that restricts the generalizability of our findings to male patients. Besides, the CTRL group of healthy volunteers was age-matched to the SjD group, but not to the NSS group, which had a lower median age. Although age-related changes in neuropeptide levels are not well defined, this remains a possible confounding factor. Moreover, several factors that may influence salivary neuropeptide levels were not systematically controlled in this preliminary study and should be considered when interpreting the results. First, smoking status could affect salivary SP levels, as cigarette smoke activates capsaicin-sensitive sensory neurons (C-fibers) and leads to the release of SP (8). Second, hormonal and menopausal status are relevant confounders, given the well-documented effects of estrogens on neuropeptide expression (10). Future larger-scale, prospective studies should systematically collect and adjust for these potential confounders to confirm the specificity and robustness of salivary SP as a potential diagnostic biomarker.

In conclusion, this pilot study provides the first evidence that salivary SP is significantly elevated in patients with SjD and can distinguish them from patients with non-autoimmune NSS. Preliminary data from this pilot study suggest that salivary SP levels could be useful as an additional non-invasive indicator in the differential diagnosis between SjD and NSS. Several avenues merit exploration in future studies. First, longitudinal validation in larger, prospective cohorts is required to confirm the stability and reproducibility of salivary SP as a biomarker across different disease stages. Second, the inclusion of multi-marker panels (for example, combining SP with other neuropeptides, cytokines, or autoantibodies) could improve diagnostic accuracy and help distinguish overlapping clinical phenotypes. Third, correlations with disease severity should be assessed to determine whether salivary SP reflects disease burden or activity. Finally, therapeutic monitoring applications should be investigated, as changes in salivary SP levels may serve as pharmacodynamic biomarkers in clinical trials targeting neuroimmune pathways in SjD.

## Data Availability

The raw data supporting the conclusions of this article will be made available by the authors, without undue reservation.
